# Discordant patterns between nitrogen-cycling functional traits and taxa in distant coastal sediments reveal important community assembly mechanisms

**DOI:** 10.3389/fmicb.2023.1291242

**Published:** 2023-11-20

**Authors:** Wen Song, Hongjun Li, Yuqi Zhou, Xia Liu, Yueyue Li, Mengqi Wang, Dan-dan Li, Qichao Tu

**Affiliations:** ^1^Institute of Marine Science and Technology, Shandong University, Qingdao, China; ^2^State Environmental Protection Key Laboratory of Coastal Ecosystem, National Marine Environmental Monitoring Center, Dalian, China

**Keywords:** nitrogen cycling, community assembly, microbial communities, functional traits, taxonomic groups, coastal sediments

## Abstract

A central question in microbial ecology is how immense microbes are assembled in changing natural environments while executing critical ecosystem functions. Over the past decade, effort has been made to unravel the contribution of stochasticity and determinism to the compositional of microbial communities. However, most studies focus on microbial taxa, ignoring the importance of functional traits. By employing shotgun metagenomic sequencing and state-of-the-art bioinformatics approaches, this study comprehensively investigated the microbially mediated nitrogen (N) cycling processes in two geographically distant coastal locations. Both shotgun and 16S rRNA gene amplicon sequencing demonstrated significantly differed taxonomic compositions between the two sites. The relative abundance of major microbial phyla, such as *Pseudomonadota*, *Thaumarchaeota*, and *Bacteroidota*, significantly differed. In contrast, high homogeneity was observed for N-cycling functional traits. Statistical analyses suggested that N-cycling taxonomic groups were more related to geographic distance, whereas microbial functional traits were more influenced by environmental factors. Multiple community assembly models demonstrated that determinism strongly governed the microbial N-cycling functional traits, whereas their carrying taxonomic groups were highly stochastic. Such discordant patterns between N-cycling functional traits and taxa demonstrated an important mechanism in microbial ecology in which essential ecosystem functions are stably maintained despite geographic distance and stochastic community assembly.

## Introduction

Nitrogen (N) is an essential element of nutrition in the Earth biosphere, making up critical life components such as proteins and nucleic acids (Kuypers et al., 2018). The regulation of N supply constrains the primary productivity of many natural ecosystems (Falkowski et al., 1998). In nature, the cycling of N is primarily driven by microorganisms, by whom N is converted among different redox states (Nelson et al., 2016), closely linked to biogeochemical cycles such as carbon, sulphur, phosphorus, and others, influencing the structure and functioning of ecosystems (Qian et al., 2023). Specifically, N-cycling microorganisms constitute an important part of biodiversity in natural ecosystems and ultimately affect ecosystem function by mediating a series of processes, including nitrogen fixation, nitrification, denitrification, ammonium oxidation, dissimilatory/assimilatory nitrate reduction to ammonium, and ammonification (Damashek and Francis, 2018). A critical question in microbial ecology is how different microorganisms are assembled into complex communities to execute these essential ecological processes.

Over the past years, much progress has been made toward our understanding of the microbially driven N-cycling processes on Earth. Novel discoveries have been continuously made, such as the discovery of complete nitrification of ammonia via nitrite to nitrate by a single microorganism (Daims et al., 2015; van Kessel et al., 2015), the regulatory and metabolic adaptations in the nitrogen assimilation of marine *Picocyanobacteria* (Díez et al., 2023), and widespread and abundant nitrogen-fixing populations of *Pseudomonadota* and *Planctomycetota* in marine and river ecosystems (Delmont et al., 2018; Behera et al., 2021b). By employing high-throughput amplicon sequencing technologies, recent studies have revealed surprisingly high genetic diversity of N-cycling marker genes in various ecosystems (Damashek and Francis, 2018; Li et al., 2023), suggesting high redundancy of N-cycling functional traits. For instance, using the Pearl River Estuary (PRE) as a study system, researchers have delineated the diversity and abundance of key microbial nitrogen cycling gene families and their carrying taxa (Wang et al., 2021). However, our understanding regarding the ecological perspectives of the whole N-cycling community remains fragmented. Questions such as how different microorganisms collaboratively maintain the stability of N-cycling function in different ecosystems require further attention.

Although high-throughput amplicon sequencing approaches provide novel insights into the genetic diversity of N-cycling gene families, technical barriers exist in cross-linking whole N-cycling processes with taxonomic compositions, limiting systematic understanding of the N-cycling pathway in complex ecosystems. Notably, technical advances in metagenomic sequencing have more or less overcome the barriers in amplicon sequencing approaches. By employing accurate functional gene databases (Tu et al., 2018; Wang et al., 2021; Mosley et al., 2022), the linkage between N-cycling microbial functional traits and taxa can be accurately and efficiently resolved (Wang et al., 2021; Song et al., 2022), providing a technological basis for disentangling the underlying ecological mechanisms at the whole pathway level.

The coastal sediments comprise a major part of the ocean ecosystem, serving as the transition zone between the terrestrial and open ocean. The high community diversity and immense biogeochemical cycling processes they mediate in coastal sediments make them an ideal place to investigate the ecological mechanisms underlying complex biogeochemical cycling processes, including N cycling. In this study, the microbial communities involved in N-cycling processes in two distant coastal sediments were investigated, aiming to address the following two ecological questions: (1) Do geographically distant coastal sediments harbor distinct N-cycling communities? (2) What ecological mechanisms mediate the diversity patterns of N-cycling communities? As previously described in other ecosystems (Nelson et al., 2016; Sunagawa et al., 2020), we expected vast diverse microbial taxa in mediating different N-cycling processes. As a critical ecosystem function, N-cycling functional traits show relatively stable distributions across space, but their carrying microbial taxa may vary greatly. Consequently, we expected the assembly of N-cycling functional traits to be highly deterministic, while the taxonomic groups would be more stochastic. The results supported our expectation and demonstrated a microbial community assembly mechanism in which essential ecosystem functions are stably maintained despite geographic distance differences and stochastic community assembly.

## Materials and methods

### Coastal sediment collection

**Figure 1:**
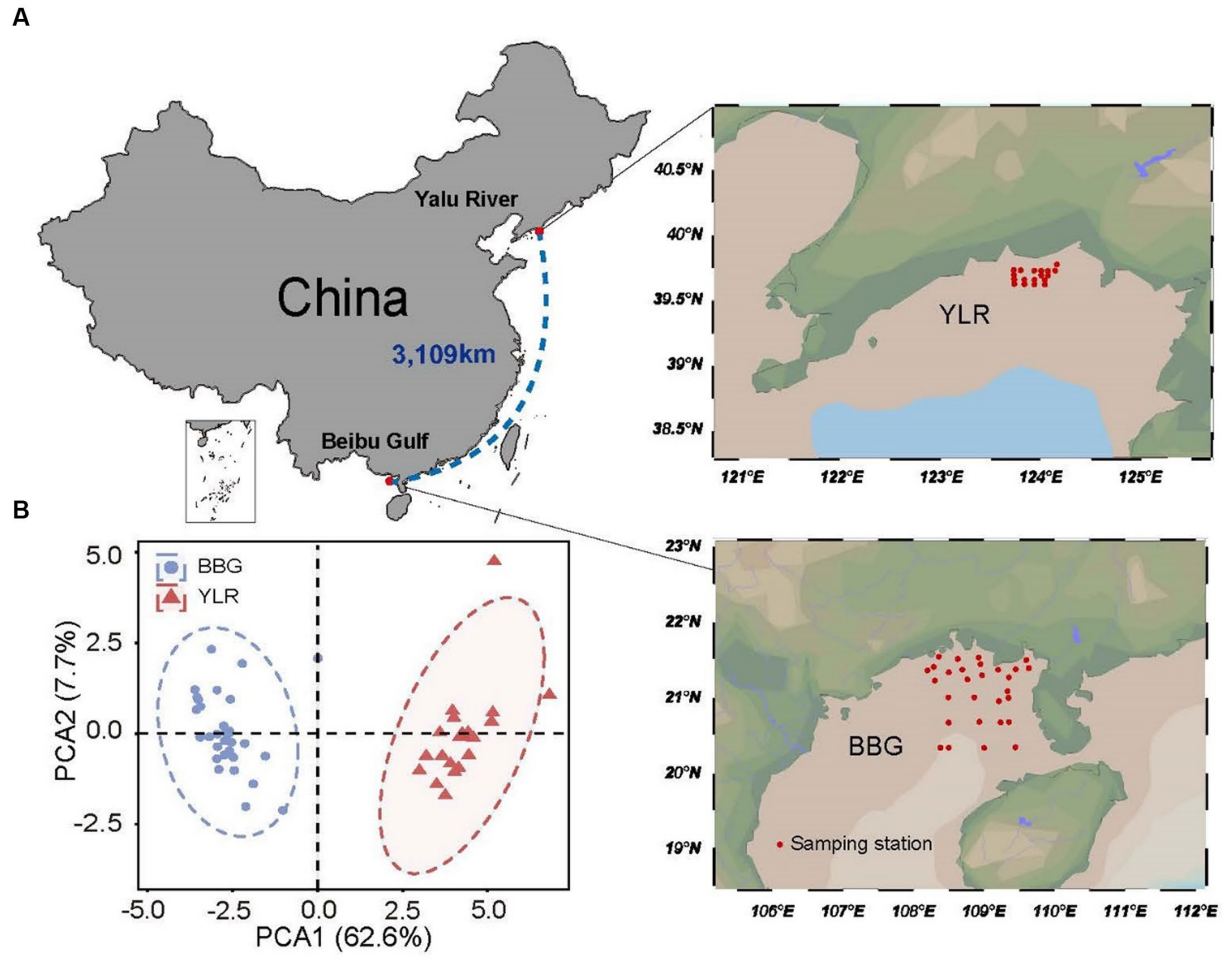
Locations of the 47 sampling stations on the temperate and subtropical coasts of China investigated in this study. **(A)** Sedimental samples were collected in two coastal sediments, including Beibu Gulf (BBG) and Yalu River (YLR). The geographic distance between the two sampling sites is 3,109 km. **(B)** Principal component analysis for environmental heterogeneity between the two sampling sites based on the environmental factor data.

Two coastal sediments were surveyed in this study, including temperate (Yalu River, YLR) and tropical (Beibu Gulf, BBG) coastal sediments in the North and South China Sea. The BBG sampling site is located at 21°07′54′′ ± 8′67′′N, 108°91′08′′ ± 8′58′′E, covering an area of approximately 16,136 km^2^. The YLR sampling site is located at 39°68′83′′ ± 1′10′′N, 123°93′14′′ ± 3′44′′E, covering an area of approximately 472.57 km^2^ (Figure 1). A total of 29 and 18 sedimental samples were collected for the YLR and BBG locations, respectively, during the cruises conducted by the National Marine Environmental Monitoring Center of Dalian on 16 July 2020 and 8 September 2020 (Supplementary Table S1). For each sampling site, three sediment replicates were collected using a van Veen grab measuring 0.05 m^2^ and subsequently combined to achieve homogenization. Each site yielded a 500 g sediment sample, which was preserved on ice and promptly transported to the laboratory within 24 h and stored at −80°C before measuring physiochemical properties and DNA extraction.

### Measuring physiochemical properties of sediment samples

The collected sediment samples were freeze-dried before analyzing their geochemical characteristics. The pH and salinity values were measured using probes (ORION STRAR A212 and EUTECH SALT 6+; Thermo Scientific, USA) with a 1:2.5 (m/v) and 1:5 (m/v) ratio of sediment to water. The concentrations of total phosphorus (TP), total nitrogen (TN), nitrate (NO_3_^−^-N), nitrite (NO_2_^−^-N), ammonium (NH_4_^+^-N), and water-soluble sulphates (SO_4_^2−^) in sediment solutions were determined by ultraviolet spectrophotometry. Sedimental NH_4_^+^-N, NO_3_^−^-N, and NO_2_^−^-N were extracted by adding 12 mL of 1 M KCl to 2 g sediment, and the sediment SO_4_^2−^ was extracted by adding 10 mL deionized water to 2 g sediment, vortexed at maximum speed for 20 min, and spun at 5,000 × *g* for 5 min. The supernatants were taken to determine the concentrations with an automated procedure (CYTATION™5; BIOTEK, USA) by measuring the absorbance at 630 nm, 410 nm, 543 nm and 420 nm (Cawse, 1967; Nelson, 1983). The DIN concentration was calculated as the sum of NH_4_^+^-N, NO_3_^−^-N and NO_2_^−^-N. The TN and TP were analyzed by weighing 2.5 g sediment, adding 35 mL alkaline K_2_S_2_O_8_ oxidizer solution, mixing well and digestion in an autoclave at 121°C for 60 min. The supernatants were taken to determine the concentrations with an automated procedure (CYTATION™5; BIOTEK, USA) to measure absorbance at 630 nm and 700 nm. The organic carbon was determined by the potassium dichromate approach (Mebius, 1960). The NH_4_^+^-N, NO_3_^−^-N, NO_2_^−^-N, SO_4_^2−^, TN, TP and petroleum hydrocarbon (PH) variables were measured following methods based on the National Standard of China (GB17378.3–2007). Moreover, inductively coupled plasma–mass spectrometry (ICP–MS, Optima, 2000 DV, PerkinElmer, USA) was used to measure the contents of seven heavy metals, including Hg, Cd, Pb, Cr, As, Cu, and Zn (Yuan et al., 2004).

### DNA extraction and sequencing

For each sample, approximately 0.5 g of thoroughly mixed sediment was used to extract genomic DNA using the DNeasy® PowerSoil Kit (QIAGEN, GERMANY, USA), following the manufacturer’s protocols. The quantity of DNA was assessed by a NanoDrop ONE (Thermo Scientific, USA) with a range of 40–60 ng/μL and stored at −80°C. An aliquot (50 ng) of purified DNA from each sample was sent to Novogene (Novogene Inc., Tianjin, China) for shotgun metagenomic sequencing and amplicon sequencing of the V3–V4 region (338F-806R) of bacterial 16S rRNA genes. The universal primers 338F (5′-ACTCCTACGGGAGGCAGCA-3′) and 806 R (5′-GGACTACHVGGGTWTCTAAT-3′) were used for PCR amplification of 16S rRNA genes. For both shotgun metagenomic and amplicon sequencing, the Illumina HiSeq PE2500 platform was used for paired-end sequencing. The 16S rRNA gene amplicon sequencing was performed for all 47 samples, while shotgun metagenome sequencing was performed for a total of 27 samples (14 for BBG and 13 for YLR).

### Metagenomic profiling of N-cycling microbial communities

In order to profile microbially mediated N-cycling processes from the shotgun metagenome sequencing data, the manually curated functional gene database NCycDB^1^ (Tu et al., 2018) was used. Briefly, shotgun metagenomic sequences were searched against NCycDB databases using DIAMOND (v 0.9.25) (Buchfink et al., 2015) in blastx mode using parameters “-k 1 -e 0.0001.” Sequences matched to NCycDB were extracted to generate functional gene profiles of N-cycling microbial communities. The Perl script in NCycDB was used to obtain functional profiles. A random subsampling procedure was applied to normalize the total number of sequences for each sample to the minimum sequencing depth. To acquire the taxonomic profiles for N-cycling microbial communities, the seqtk program was used to extract the sequences mapped by NCycDB. Taxonomic assignment was then carried out using Kraken 2 (Wood et al., 2019). A taxonomic profile was generated for the N-cycling pathway at various taxonomic levels using a standard local Kraken2 database. In this study, we defined each microbially mediated N-cycling gene family as one functional trait for mediating a specific process/function (Violle et al., 2007), whereas the corresponding microbial species as taxa. The abundance of each N-cycling gene family was calculated as the proportion of each nitrogen cycling gene to the total number of all nitrogen cycling genes in each sample. The total number of sequences referred to all N-cycling gene related reads.

### 16S rRNA gene amplicon sequencing analysis

The 16S rRNA gene amplicon sequence data were processed using the DADA2 pipeline (Callahan et al., 2016). The raw data was first demultiplexed according to the barcode, followed by primer removal. Longer sequences were obtained by merging forward and reverse reads for subsequent analysis. Chimeras were identified and removed. Then, these sequences were filtered to remove chimeras and sequences of nonbacterial origin. These sequences were further clustered into amplicon sequence variants (ASVs), resulting in 15,735 ASVs. The ASV abundance profile was then subjected to a random subsampling effort of 7,944 per sample. Taxonomy assignment was carried out against the Ribosomal Database Project (RDP) 16S rRNA gene training set online database^2^ (Wang et al., 2007). The default confidence interval cutoff of 80% was used for the RDP classifier.

### MiTAG profiling of microbial communities

In addition to 16S rRNA gene amplicons, the miTAG approach (Logares et al., 2014) was also employed to profile bacterial community composition from metagenome sequences, providing complementary information to 16S rRNA gene amplicon data without potential bias caused by PCR amplification. Here, SortMeRNA (Kopylova et al., 2012) was used to extract 16S rRNA sequences from shotgun metagenomes by searching the metagenomic reads against the Silva SSU database (V138) (Yilmaz et al., 2014). Reads matching the Silva SSU database with an e-value <10^−4^ were further filtered with custom Perl and R scripts. A total of 88,987 MiTAG sequences were recovered from the shotgun metagenomes, resulting in 7,490 sequences per sample. The RDP classifier was then employed for taxonomic assignment of the extracted sequences. Microbial profiles were generated at different taxonomic levels.

### Statistical analyses of community diversity and structure

The non-metric multidimensional scaling (NMDS) ordination was performed to explore the compositional differences in microbial functional traits and taxonomic groups between BBG and YLR. The Bray–Curtis dissimilarity index was employed to quantify the differences among different samples. Response ratio analysis (Lajeunesse, 2011) was employed to evaluate the differences in the relative abundance of N-cycling microbial functional traits and taxonomic groups present in BBG and YLR sediments.

$$RR=\ln\left(\frac{{\bar{X}}_{B}}{{\bar{X}}_{Y}}\right)=\ln\left({\bar{X}}_{B}\right)-\ln\left({\bar{X}}_{Y}\right)$$

where RR is the natural-log proportional change in the mean relative abundance ( $\bar{X}$ ) of BBG and YLR sediments.

### Correlating environmental factors with N-cycling communities

Multiple statistical tests were conducted to investigate the potential environmental factors that affect the variations in the composition and diversity of N-cycling functional traits and taxa. First, the effects of environmental factors on N-cycling functional traits and taxa were evaluated using the partial Mantel test. The distance matrices for N-cycling functional traits and taxa were, respectively, correlated with environmental parameters. Second, to determine the potential correlation between community similarity and environmental heterogeneity, a thorough assessment was conducted. The environmental factors that we used to calculate environmental distance included temperature, pH, salinity, NH_4_^+^-N, NO_3_^−^-N, NO_2_^−^-N, SO_4_^2−^, TN, TP, petroleum hydrocarbons (PHs), organic carbon (OC), organic matter (OM), Hg, Cd, Pb, Cr, As, Cu, and Zn. Here, the pairwise environment distances between different samples were calculated using Euclidean distance. The Bray–Curtis community similarity of N-cycling communities (functional traits and taxa) was correlated with the Euclidean distance of environmental conditions based on Spearman’s rank correlations. Third, to include potential nonlinear relationships and multivariate interactions, a random-forest machine-learning approach was used to estimate the important predictors for both N-cycling functional traits and taxonomic groups (Liaw and Wiener, 2002). The relative importance of environmental factors in explaining the compositional differences in N-cycling microbial functional traits and taxa (phylum level) were investigated. To conduct the above analyses, R packages such as “vegan,” “randomForest,” and “relaimpo” were used.

### Inferring community assembly mechanisms

In addition to investigating the effects of environmental factors on N-cycling communities, we also compared the relative contributions of deterministic and stochastic processes to N-cycling processes. Three complementary analyses were carried out for N-cycling microbial functional traits and taxa. First, variation partitioning analysis (VPA) was employed to evaluate the relative importance of environmental factors and geographic distance in shaping the compositional variations in N-cycling communities. We calculated spatial variables based on the longitude and latitude coordinates of each sampling station using the principal coordinates of neighbor matrices (PCNMs) (Lear et al., 2014; Righetti et al., 2019). The canonical correlation analysis (CCA) model was employed to select spatial and environmental variables using a forward selection procedure. VPA factors were chosen based on significance levels (*p* < 0.05) until no improvement in VPA was observed with additional new variables. After selection based on forward selection and Arch Effect *p*-values. The final VPA model includes PCNM14, PCNM16, PCNM18, PCNM24, pH, salinity, NH_4_^+^-N, SO_4_^2−^, TN, TP, Zn, Hg, and Cr. Second, a null-model approach was used to quantify the relative importance of deterministic vs. stochastic processes. Using Bray–Curtis distance, a null distribution of beta diversity was obtained by shuffling the original communities randomly (1,000 randomizations), on the basis of which we calculated the normalized stochasticity ratio (NST) (Ning et al., 2019). During the assembly process, a ratio of 50% was chosen as the cutoff to distinguish between more deterministic (50% or less) and more stochastic assembly (over 50%) processes. Third, in addition to the null-model approach, a neutral community model (NCM) was also employed to determine the potential importance of stochastic processes on community assembly (Sloan et al., 2006). It was predicted that species frequency and relative abundance across the wider metacommunity would be correlated in the NCM. The parameter R^2^ represents the overall fit to the neutral model within the 95% confidence interval of the NCM predictions. R packages including vegan (e.g., pcnm, bioenv), NST, and tidyverse were used for the above analyses. All statistical analyses were performed in R software (v.4.3.2) (Oksanen et al., 2013).

## Results

### Overall community characteristics in BBG and YLR sediments

A total of 29 and 18 sediment samples were collected from the BBG and YLR sampling sites, respectively. These two sampling sites were geographically distant (3,109 km) and distinctly different in environmental conditions (Figures 1A,B). All samples were subjected to 16S rRNA gene amplicon sequencing, while 27 of them (13 from BBG and 14 from YLR) were subjected to shotgun metagenomic sequencing (Supplementary Table S2). For each sample, an average of 53,780 high-quality 16S rRNA sequences and 20 Gb shotgun metagenome sequences were obtained. Random subsampling of 7,944 16S rRNA gene sequences resulted in 2,903 and 9,221 ASVs per sample for BBG and YLR, respectively. In addition, these two sampling sites were found to have distinctly different microbial compositions, as revealed by both 16S rRNA gene amplicon sequencing and 16S rRNA gene extraction from shotgun metagenomes (i.e., miTAG microbial profiles, Supplementary Figure S1).

**Figure 2:**
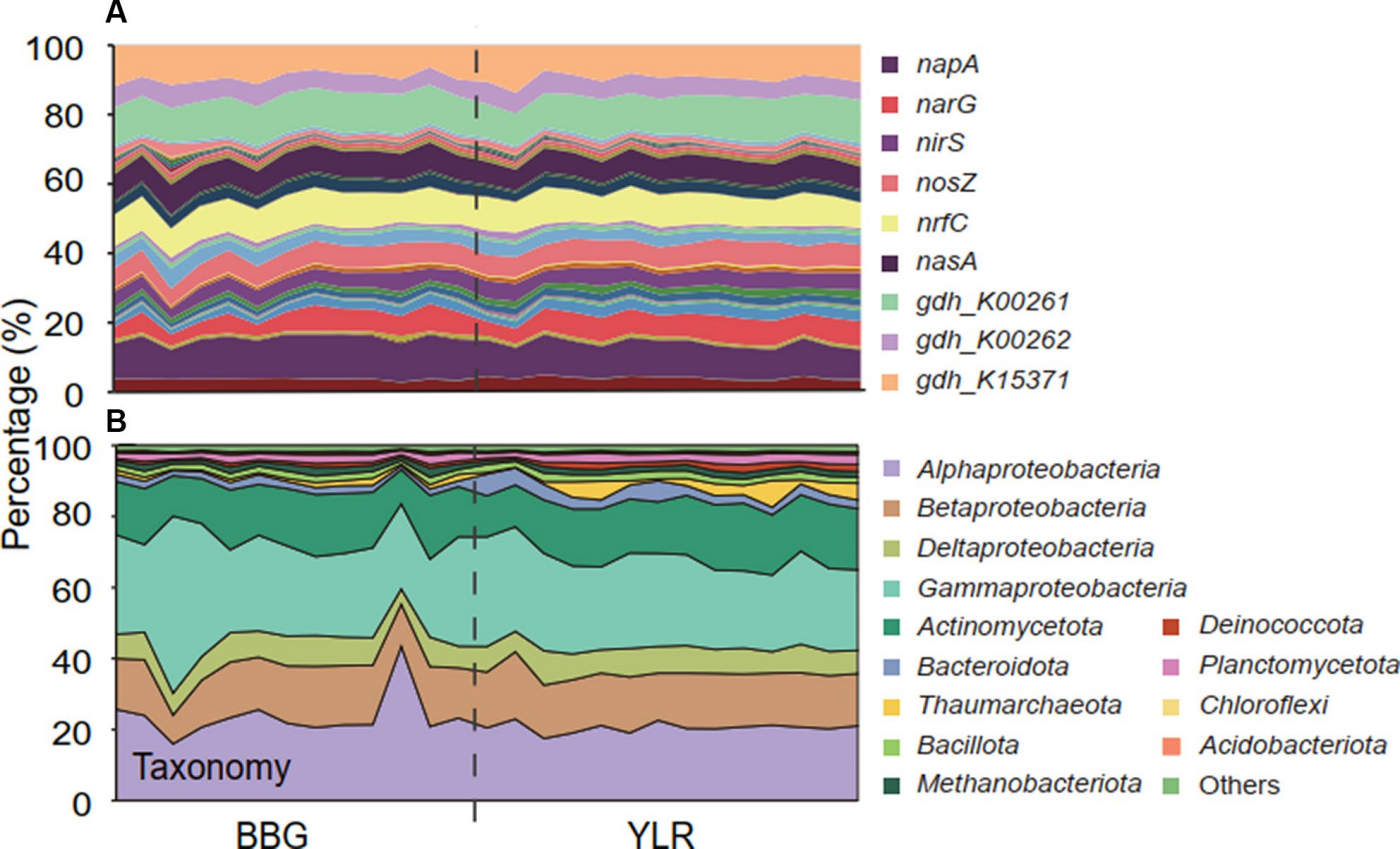
The composition of microbial N-cycling functional traits and taxa (phylum-level, class-level for *Pseudomonadota*) in BBG and YLR. **(A)** Relative abundances of microbial N-cycling functional traits. Color codes were displayed for the top 9 most abundant gene families. Supplementary Table S2 contains the exact relative abundance and corresponding color for each functional trait. **(B)** Relative abundances of different taxonomic groups.

Interestingly, distinct microbial taxa carried homogeneous N-cycling functional traits. Microbial communities involved in N-cycling processes were extracted by searching shotgun metagenomes against NCycDB (Tu et al., 2018), a manually curated functional gene database targeting N-cycling pathways. An average of 131,752,546 sequences were found to be N-cycling genes in each sample, occupying 0.54% of the total sequences. Taxonomic assignment of these N-cycling genes using Kraken2 showed an assignment rate of 38.7% to currently known microbial taxa, suggesting that a high portion of N-cycling genes were still taxonomically unknown. In total, 53 N-cycling functional traits and 5,588 taxonomic groups were found to be present in at least one sample. The relative abundances of functional traits were highly similar and compositionally not distinguishable (Figure 2A). In contrast, consistent with the whole community level, a significantly different taxonomic composition was observed for the N-cycling communities (Figure 2B).

**Figure 3:**
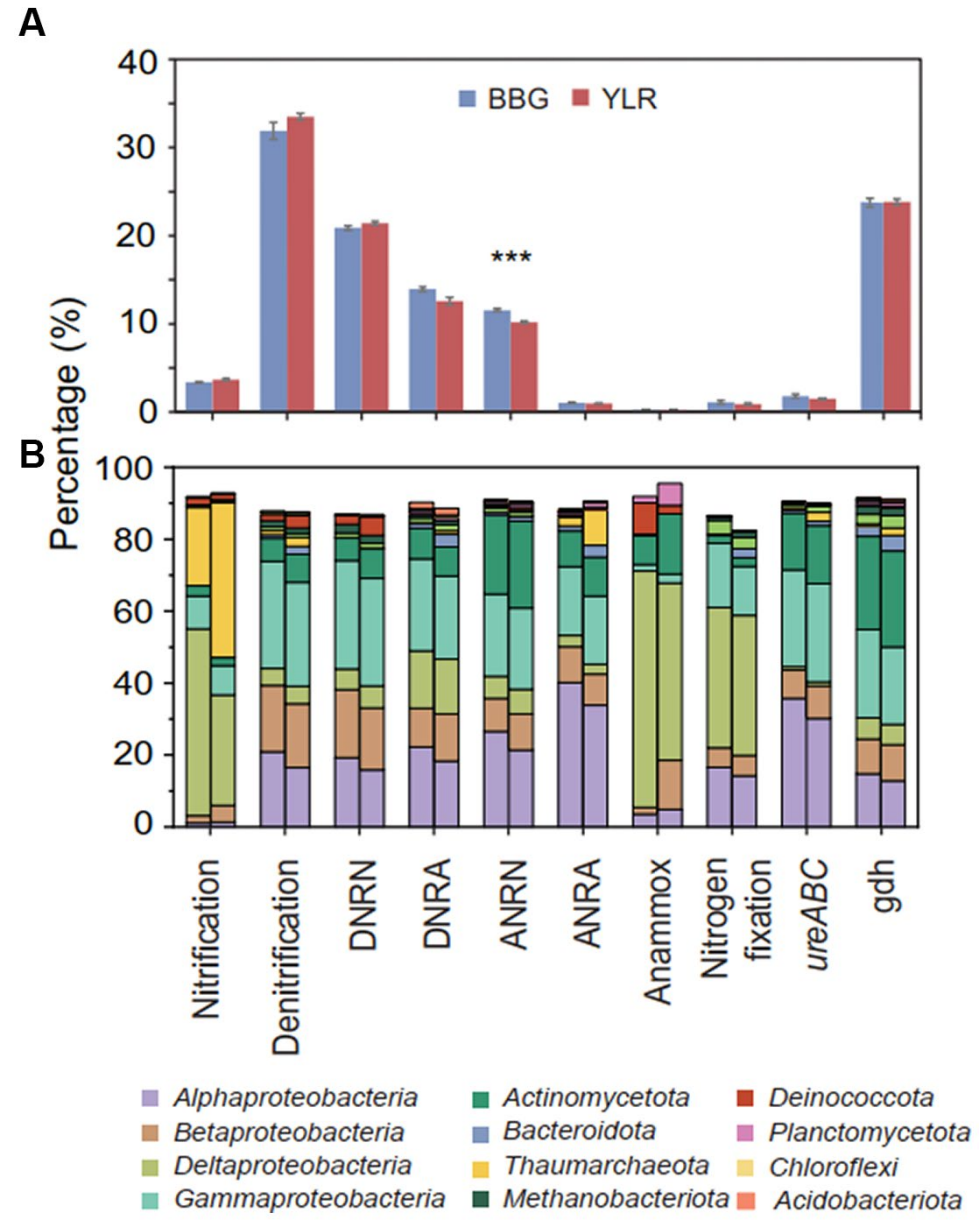
**(A)** Relative abundances of different N-cycling pathways. Significant differences between the two sampling sites were displayed by asterisks (***p < 0.001). **(B)** The left (BBG) and right (YLR) bars represent the average relative abundance of N-cycling microbial communities at the phylum (top 10 in abundance) and class (top 4 in abundance) levels. Different N-cycling pathways were shown. DNRN, dissimilatory nitrate reduction to nitrite; DNRA, dissimilatory nitrite reduction to ammonia; ANRN, assimilatory nitrate reduction to nitrite; ANRA, assimilatory nitrite reduction to ammonia.

Functionally, microbial functional traits mediating N-cycling processes were relatively evenly and stably distributed across different sampling sites in BBG and YLR, regardless of their locations (Figures 2A, 3A). Among the various N-cycling processes, only assimilatory nitrate reduction to nitrite (ANRN) showed significantly differed relative abundances between BBG and YLR (Figure 3A). Consequently, gene families including *nasA*, *nasB* and *narB* were more abundant in BBG (Supplementary Table S2). This suggested that the southern sampling site BBG could be more functional in assimilatory conversion of ammonia into nitrite. Bacterial *amo* gene families were more abundantly detected in the YLR sediment than in the BBG sediment, suggesting that AOB taxa may favor lower temperature in the ocean sediment (Supplementary Table S2). In addition, gene families including *napA*, *narHIZ*, *nirK*, and *norC* involved in denitrification and DNRN (dissimilatory nitrate reduction to nitrite) also showed significant differences in relative abundance between BBG and YLR (Supplementary Table S2). The different patterns in the relative abundance of *nap* (e.g., *napAB*) and *nar* (e.g., *narHIZ*) between BBG and YLR suggested that these two sites favored different gene families in dissimilatory nitrate reduction to nitrite. In both BBG and YLR, the relative abundances of archaeal *amo* gene families were all significantly higher than those of bacterial *amo* gene families (Supplementary Figure S2A), suggesting that archaea rather than bacteria dominated ammonia oxidization to hydroxylamine in the investigated coastal sediments.

Taxonomically, much more complex information was observed than functional traits. First, the N-cycling communities were dramatically different from the whole prokaryotic communities, based on either the 16S amplicon or miTAG results (Figure 2B; Supplementary Figure S3). Taxonomic assignment against the RDP database identified a total of 54 phyla for the MiTAG dataset. In contrast, for the 16S rRNA gene amplicon dataset, 36 phyla were identified. Not surprisingly, these two different approaches generated substantially different microbial profiles, even at the phylum level (Supplementary Figure S3). For instance, the relative abundance for the major phyla dramatically differed using these two approaches, which was especially evident for the YLR samples (Supplementary Figure S3). Nonetheless, both approaches demonstrated dramatically different community compositions between BBG and YLR. *Actinomycetota* was overall more abundantly detected in YLR than in BBW, but with a similarly high contribution to N-cycling processes. Microbial taxa belonging to *Acidobacteriota* and *Chloroflexota* were rarely found in N-cycling communities but were abundantly observed in the sediments. *Pseudomonadota* and *Actinomycetota* dominated the N-cycling communities at both sites, contributing 65.9–84.4% and 9.1–17.8% of the relative abundance, respectively (Figure 2B). *Pseudomonadota* taxa were significantly more abundant in BBG, whereas *Bacteroidota*, *Thaumarchaeota*, and *Methanobacteriota* taxa were more abundant in YLR (Figure 2B; Supplementary Figure S4). Of these, *Thaumarchaeota* was mainly detected in nitrification (21.8% in BBG and 43.1% in YLR) and assimilatory nitrite reduction to ammonia (ANRA) (2.52% in BBG and 9.75% in YLR, Figure 2D). Second, different N-cycling processes and functional traits were mediated by highly different microbial taxonomic groups (Figure 3B; Supplementary Figure S2B). For instance, nitrification, the process that converts ammonia into nitrate, was mainly mediated by *Deltaproteobacteria* and *Thaumarchaeota* in this study. Anammox, which converts ammonium into dinitrogen and was low in relative abundance, was mainly mediated by *Deltaproteobacteria* and *Actinomycetota*. Almost all denitrification gene families were dominated by *Pseudomonadota* (71 to 98.4%), except *nirK*, for which *Thaumarchaeota* (49.5%) was the dominant phylum. Third, different N-cycling processes were generally similar in relative abundance between BBG and YLR. However, their taxonomic composition between BBG and YLR was considerably different (Figure 3B). For instance, the relative contribution of *Deltaproteobacteria* and *Thaumarchaeota* to nitrification dramatically differed between BBG and YLR. For anammox, in addition to the major and different contributions of *Deltaproteobacteria* and *Actinomycetota* to anammox in BBG and YLR, BBG was also enriched by *Methanobacteriota*, whereas YLR was enriched by *Planctomycetota* and *Betaproteobacteria*. Such results suggested that geographically distant coastal sediments were similar in N-cycling functional traits but dramatically different in their carrying microbial taxa.

### Environmental drivers of sedimental N-cycling communities

**Figure 4:**
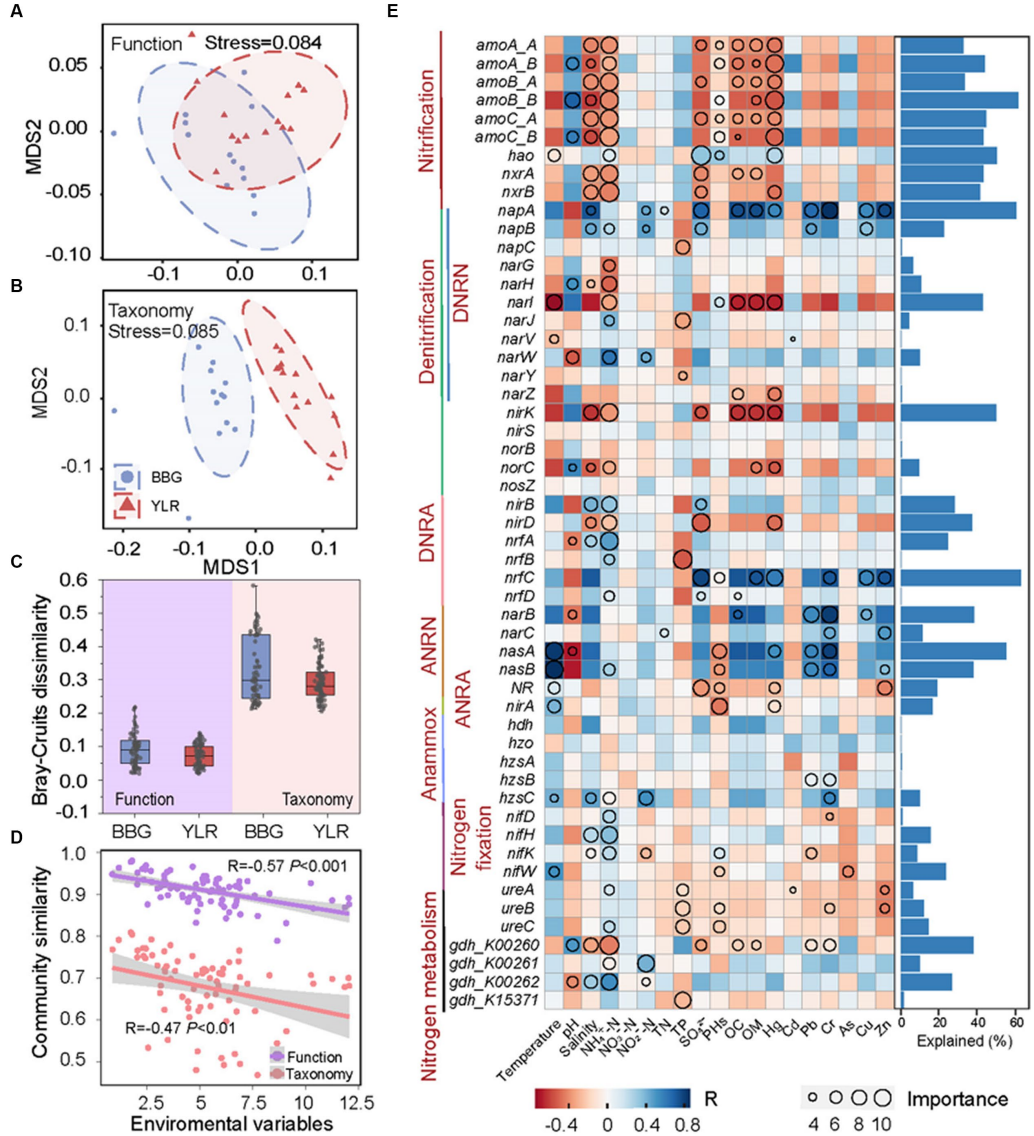
Relationship between N-cycling communities and environmental factors in BBG and YLR sampling sites. In order to assess the distance compositional variation of N-cycling functional trait **(A)** and taxa **(B)**, a non-metric multidimensional scaling (NMDS) was applied based on Bray–Curtis dissimilarity. **(C)** Bray–Curtis dissimilarity of N-cycling functional trait and taxa in BBG and YLR. **(D)** Spearman’s rank correlations between the Bray–Curtis similarity of N-cycling communities and the Euclidean distance of environmental variables. **(E)** Explainable variations by different environmental factors for different functional traits. As indicated by the heatmap (on the left panel), environmental factors were important in explaining the variations in functional traits based on random forest analysis, whereas the circle size indicated the degree to which environmental factors played a role. In the right panel, bar plots represent the variations explained by the best environmental factor (the circle with the largest diameter). DNRN, dissimilatory nitrate reduction to nitrite; DNRA, dissimilatory nitrite reduction to ammonia; ANRN, assimilatory nitrate reduction to nitrite; ANRA, assimilatory nitrite reduction to ammonia.

Dramatically different diversity patterns in composition were observed between the N-cycling functional traits and their carrying microbial taxonomy. First, the taxonomic composition of N-cycling microbial communities between BBG and YLR sediments was clearly different, whereas the structure of N-cycling microbial functional traits was compositionally not distinguishable (Figures 4A,B). Second, for both BBG and YLR, functional traits showed much lower between-sample community similarity than taxonomic groups (Figure 4C). We therefore expected different effects of environmental conditions on N-cycling functional traits and taxonomy.

We first investigated the overall influence of environmental variables on N-cycling communities. Here, the Euclidean distance of 19 environmental conditions was calculated as a measurement of environmental heterogeneity and was correlated with N-cycling community similarity based on functional traits and taxonomic groups. Strong negative associations between environmental heterogeneity and N-cycling community similarity were observed (Figure 4D). Notably, a stronger association was observed between environmental heterogeneity and N-cycling functional traits (*R* = −0.57, *p* < 0.001) than taxonomic groups (*R* = −0.47, *p* < 0.01). In addition, the partial Mantel test by sequentially excluding individual environmental parameters showed that four environmental factors were significantly associated with the N-cycling taxonomic composition, while the number of significantly associated environmental parameters was eight for functional traits (Supplementary Table S3). This suggested that environmental heterogeneity exerted a stronger influence on the compositional difference of N-cycling functional traits than taxonomic groups, although N-cycling functional traits harbored weaker compositional variation than microbial taxa. The influence of environmental factors on individual N-cycling functional traits and taxonomic groups was also investigated using a multiple stepwise regression model and a machine learning approach random forest. As a result, multiple N-cycling functional traits can be well explained by one or more environmental factors (Figure 4E). The effects of different environmental variables on the functional traits belonging to the same process were generally similar, suggesting that microorganisms performing the same function tended to be involved in the same ecological niche. For instance, functional traits involved in nitrification were strongly associated with salinity, NH_4_^+^-N, and Hg, with explanation rates larger than 40%. In addition, functional traits involved in ANRN were strongly associated with heavy metal contamination, such as Pb and Cr. Among various environmental factors, NH_4_^+^-N was the major factor affecting multiple functional traits involved in different processes. In contrast, the association between environmental factors and taxonomic groups was relatively weak, especially for abundant phyla. Notably, *Thaumarchaeota* and *Methanobacteriota*, which are two representative phyla involved in nitrification and anammox, were best explained by NH_4_^+^-N (Supplementary Figure S5). This was consistent with the fact that both nitrification and anammox were intermediated with ammonia.

### Community assembly mechanisms for N-cycling communities

**Figure 5:**
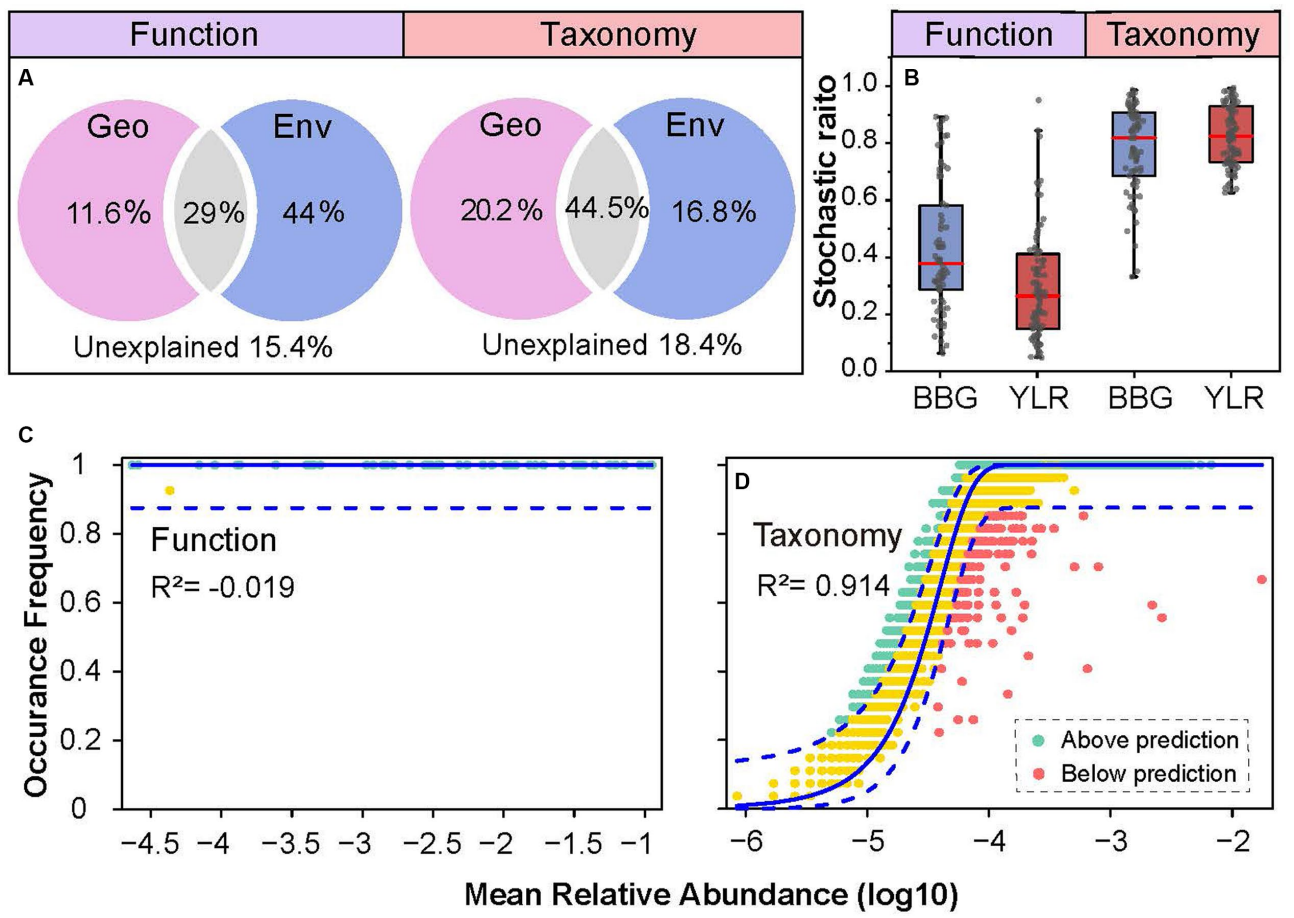
Community assembly mechanisms governing N-cycling microbial functional traits and taxa. **(A)** Contribution of geo-environmental factors to N-cycling microbial taxa and functional traits as revealed by variation partitioning analysis. **(B)** Stochastic ratio analyses using a null model showing the relative contribution of stochastic and deterministic processes in the assembly of N-cycling communities. **(C,D)** NCM analyses of community assembly for N-cycling functional traits **(C)** and taxonomic groups **(D)**. The predicted occurrence frequencies for N-cycling functional traits and taxonomic groups communities were analyzed. The model prediction has 95% confidence intervals around it, represented by the dashed blue lines. Community compositions that occurred more or less frequently than predicted by the NCM are shown in different colors.

Both deterministic and stochastic processes shape the compositional variations of complex microbial communities. Here, multiple approaches were used to quantify the role that deterministic and stochastic processes play in shaping the functional and taxonomic composition of the sedimental N-cycling communities. First, the contributions of geographic distance and environmental variables in explaining N-cycling communities were quantified (Figure 5A). The total explained ratios by geographic distance and environmental variables were similar for N-cycling microbial functional traits (84.6%) and taxa (81.6%). However, the ratios of geographic distance and environmental variables were dramatically different. The pure explanation ratio of environmental variables was 44% for functional trait composition, but was only 16.8% for taxonomic composition. This indicated that N-cycling functional traits were more strongly affected by environmental conditions, consistent with the above results obtained by partial Mantel tests and random forest modeling. Second, which is consistent with the VPA results, analyses of stochastic ratios indicated a high degree of determinism in the assembly of N-cycling functional traits (0.36), whereas the assembly of N-cycling taxonomic groups was more stochastic (0.81, Figure 5B). Third, the NCM was used to estimate and predict the frequency distribution of N-cycling functional traits and taxonomic groups. The frequency of N-cycling functional traits was poorly predicted by the NCM (Figure 5C). In contrast, the frequency of microbial taxonomic groups that occurred in communities was well described by the NCM, explaining as much as 91.4% of compositional variations (Figure 5D), suggesting that neutral processes governed the frequency and occurrence of N-cycling taxonomic groups. Integrating all of the above lines of evidence, the results demonstrated that N-cycling functional traits were highly deterministic, whereas their carrying microbial taxa were relatively stochastic.

## Discussion

### Microbial communities mediating N-cycling processes

Linking microbial function with taxonomy has been challenging in the field of microbial ecology due to the vast diversity and unknown majority of microbial taxa in natural ecosystems (Fierer, 2017). The emerging shotgun metaghenome sequencing technology has been routinely used to elucidate the structure, function, and ecology of microbiota in evolving climates and ecosystems (Behera et al., 2021a,b). In this study, by employing shotgun metagenome sequencing and the current state-of-the-art curated functional gene database (NCycDB) (Tu et al., 2018), the diversity and compositional variations of microbial communities responsible for N cycling were investigated in two geographically distant coastal sediments. Comparative analyses were conducted from two different perspectives, including functional traits and taxonomic groups. Several interesting findings were obtained. First, despite the fact that different techniques generate different microbial profiles (e.g., 16S amplicon vs. miTAG) (Logares et al., 2012, 2014). However, the taxonomic composition of N-cycling communities was dramatically different from that of the whole microbial communities. Similar findings were obtained from other ecosystems, such as the Ganges ecosystem (Parida et al., 2022), suggesting that N-cycling processes occurred in selective microbial taxa (Strom, 2008). Second, although lowly detected in the overall communities, microbial phyla such as *Bacteroidota*, *Thaumarchaeota*, and *Methanobacteriota* played critical roles in N-cycling and were significantly enriched in YLR sediments, especially *Thaumarchaeota*, which was found to be the major phyla responsible for nitrification and nitrite reduction by *nirK* (Reji et al., 2019). Such results suggested that rare and less abundant microbial taxa may have executed critical ecosystem functions in natural environments (Jousset et al., 2017). Third, consistent with several previous studies (He et al., 2012; Kozlowski et al., 2016; Kitzinger et al., 2019), archaea (more specifically *Thaumarchaeota*) rather than bacteria dominated the process of ammonia oxidization to hydroxylamine. All these results demonstrated that whole N cycling is a result of collaborative effort by the vast diverse microbial taxa in the ecosystem (Huber et al., 2007; Nelson et al., 2016).

### Discordant patterns between N-cycling functional traits and taxonomy

Divergent trends were identified for the N-cycling functional traits and carrying taxa in the BBG and YLR sediments. Consistent with our expectation, N-cycling functional traits were relatively stably distributed in the two geographically distant coastal sediments, while their carrying microbial taxa dramatically varied. Similar discordant patterns between microbial functional traits and taxonomy have also been observed at much larger scales in different ecosystems, e.g., the global ocean (Sunagawa et al., 2015; Song et al., 2022), global soil (Bahram et al., 2018), polluted river sediment (Behera et al., 2021b; Rout et al., 2022), and human microbiome (Tian et al., 2020). Different microbial ecological principles underlie this phenomenon. First, thousands of different microbial taxa may carry the same functional trait, as revealed by amplicon sequencing studies (Bru et al., 2011; Tu et al., 2016), resulting in high functional redundancy in microbial systems (Allison and Martiny, 2008; Louca et al., 2018; Tian et al., 2020). Therefore, the taxonomic composition of microbial communities executing the same function may vary. Second, the ecosystem selects microbial functional traits rather than taxonomy (Burke et al., 2011). Although species are the basic unit of the life forms in the Earth’s biosphere and directly interact with the environment, this study, together with several others (Stilianos et al., 2016; Escalas et al., 2019), suggests that the functional traits carried by microbial taxa are more important in the ecosystem. Variations in microbial taxonomic composition are constrained by the ecosystem to maintain essential ecosystem function, such as N-cycling. Therefore, functional redundancy and ecosystem selection of functional traits jointly shape the variation in N-cycling taxonomic composition and maintain the stability of functional traits.

### Perspectives for ecological mechanisms driving microbial community assembly

One of the most critical questions in microbial ecology is how such complex microbial assemblages, consisting of thousands of different taxa, are formed in natural ecosystems (DeLong et al., 2006; Burke et al., 2011; Behera et al., 2021a), performing crucial ecosystem functions and maintaining ecosystem equilibrium. Over the past decade, machine learning algorithms have been conducted by researchers to categorize newly identified and unidentified microorganisms in metagenomic datasets (Choudhury et al., 2023). In addition, many studies have been carried out to disentangle the mechanisms governing complex microbial community assembly (Stegen et al., 2015; Zhou and Ning, 2017; Wu et al., 2023). A general consensus was that microbial community composition is shaped by a combination of deterministic and stochastic processes (Stegen et al., 2012; Tripathi et al., 2018). Notably, previous studies solely rely on microbial taxonomic composition to infer the relative importance of stochastic vs. deterministic processes (Chen et al., 2019; Huang et al., 2022), ignoring the functional traits carried by microbial taxa. In this study, the relative importance of stochastic vs. deterministic processes was investigated from both taxonomic and functional trait angles. Similar to what has been observed in the global ocean (Song et al., 2022), multiple lines of empirical evidence have indicated that the composition of microbial N-cycling functional traits is predominantly governed by deterministic factors, while N-cycling microbial taxa exhibit a high degree of stochasticity.

Taking together the above evidence and prior knowledge in microbial systems (Goldford et al., 2018), we propose the following explanation for how microbial communities are assembled in natural ecosystems. Essential ecosystem functions are executed and maintained by various microbial taxa in natural ecosystems. Due to functional redundancy in microbial systems (Louca et al., 2018; Biggs et al., 2020), the same functional traits may be carried by many different microbial taxa. Only the microbial taxa executing the required functions and adapting to the environmental conditions were selected by the ecosystem. For instance, despite the extensive release of varied polluted wastewater into the Ganges, abundant phages executing similar functions actively combat harmful pathogens, preserving the river’s self-purification capability (Behera et al., 2023). Therefore, microbial functional traits are more important than taxa, especially for functionally redundant communities. The formation of complex microbial assemblages is constrained to guarantee ecosystem multifunctioning and stability. Consequently, the microbial functional trait composition is strongly deterministic, while the taxonomic composition is more stochastic. However, the stochasticity of microbial taxonomic composition may depend on environmental conditions. In light of this, we propose that the ecosystem prioritizes the selection of microbial functional traits over taxonomy, with functional redundancy serving as the foundation for the stochastic composition of microbial taxa.

## Conclusion

In this study, the microbially mediated N-cycling processes in two geographically distant coastal sediments were characterized using shotgun metagenome sequencing approaches. By employing state-of-the-art bioinformatics approaches, the composition and diversity patterns of N-cycling microbial functional traits and their carrying taxa were comparatively investigated. Discordant patterns were observed between N-cycling functional traits and taxa. N-cycling functional traits were highly homogeneous between the two distant coastal sediments, while their carrying microbial taxa varied greatly. Further investigation suggested that the compositional variations in N-cycling functional traits were more affected by environmental factors and subjected to deterministic processes. In contrast, the differences in the composition of microbial taxa carrying N-cycling functional traits were highly stochastic. Additionally, this study reveals basic information regarding N-cycling processes mediated by microbes as well as demonstrating an essential ecological mechanism explaining the assembly of complex microbial community assembly in natural ecosystems, whose essential ecosystem functions are maintained despite of geographic distance and stochastic community assembly.

## Data availability statement

Sequencing data generated in this study is deposited at the NCBI SRA portal under project ID PRJNA842863 and PRJNA857996, as well as at NODE (https://www.biosino.org/node/) under project ID OEP003498.

## Author contributions

WS: Conceptualization, Data curation, Formal analysis, Investigation, Methodology, Writing – original draft. HL: Data curation, Writing – review & editing. YZ: Data curation, Methodology, Writing – review & editing. XL: Methodology, Writing – review & editing. YL: Data curation, Methodology, Writing – review & editing. MW: Investigation, Methodology, Writing – review & editing. D-dL: Writing – review & editing. QT: Conceptualization, Funding acquisition, Project administration, Writing – review & editing.

## Footnotes

^1^ https://github.com/qichao1984/NCyc

^2^ http://rdp.cme.msu.edu/classifier/classifier.jsp
