# Supplementary material for: Discordant patterns between nitrogen-cycling functional traits and taxa in distant coastal sediments reveal important community assembly mechanisms
Source: Front Microbiol. 2023 Nov 20;14:1291242. doi: 10.3389/fmicb.2023.1291242 (PMC10694361; doi:10.3389/fmicb.2023.1291242)
Supplement: Supplementary file 3 [file Data_Sheet_1.docx]

Supplementary Material

**Discordant patterns between nitrogen-cycling functional traits and taxa in distant coastal sediments reveal important community assembly mechanisms**

Wen Song ^1^, Hongjun Li ^2^, Yuqi Zhou ^1^, Xia Liu ^1^, Yueyue Li ^1^, Mengqi Wang ^1^, Qichao Tu ^1*^

^1.^ Institute of Marine Science and Technology, Shandong University, Qingdao, China

^2.^ State Environmental Protection Key Laboratory of Coastal Ecosystem, National Marine Environmental Monitoring Center, Dalian, China

^*^ To whom correspondence should be addressed: tuqichao@sdu.edu.cn

1. **Supplementary figures**

**Supplementary Figure 1.** Ordination of the composition of (A) 16S rRNA and (B) miTAG using the non-metric multidimensional scaling based on Bray-Curtis dissimilarity.

**Supplementary Figure 2.** The mean relative abundance and response ratio of the N-cycling functional traits between BBG and YLR.

**Supplementary Figure 3.** Relative abundances of microbial phyla involved in denitrification pathway and functional traits responsible for ammonification in BBG and YLR.

**Supplementary Figure 4.** Relative abundances of 16S rRNA and miTAG on phylum-level microbial communities between BBG and YLR.

**Supplementary Figure 5.** The mean relative abundance and response ratio of microbial phyla (class-level for *Pseudomonadota*) between in BBG and YLR.

**Supplementary Figure 6.** The association of environmental factors and N-cycling microbial communities in BBG and YLR.

**B.** **Supplementary tables**

**Supplementary Table 1** Relative abundance of microbial functional traits involved in N-cycling processes in BBG and YLR.

**Supplementary Table 2** The partial Mantel test describes the effects of environmental factors on the microbial composition of N-cycling functional traits and taxonomic groups between BBG and YLR.

1. **Supplementary Figures**


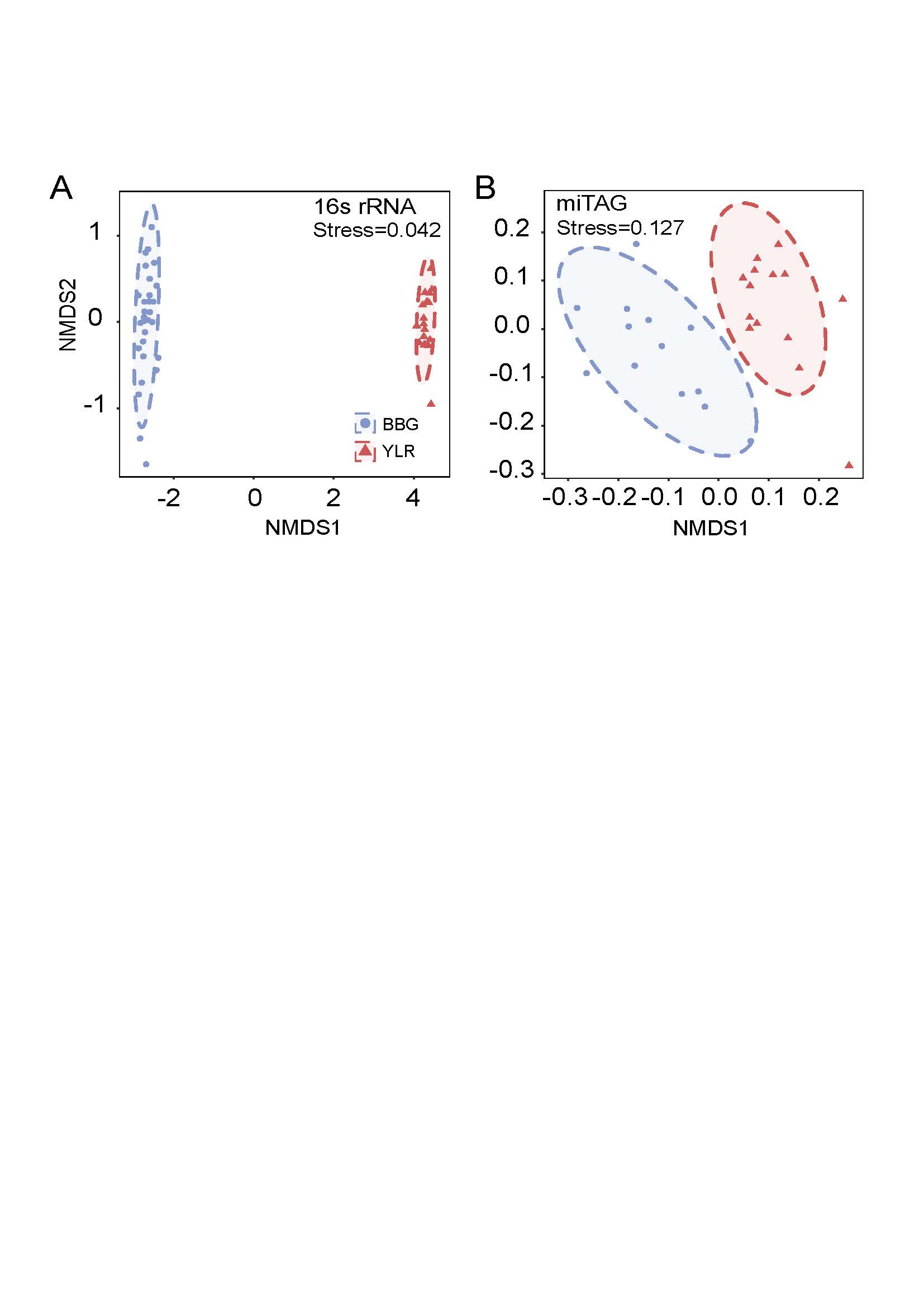


Supplementary Figure 1. Ordination analyses of the composition of (A) 16S rRNA and (B) miTAG using the non-metric multidimensional scaling based on Bray-Curtis dissimilarity.


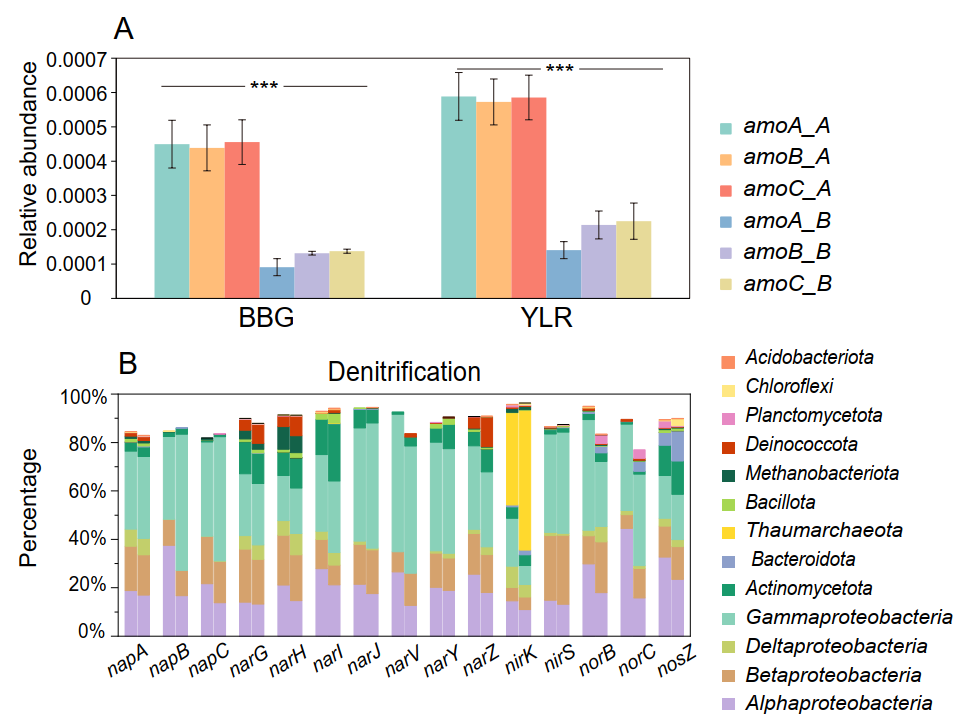


Supplementary Figure 2. (A) Relative abundances of functional traits responsible for ammonification and relative abundances of microbial phyla (class-level for *Pseudomonadota*) involved in denitrification pathway (B) in BBG and YLR.


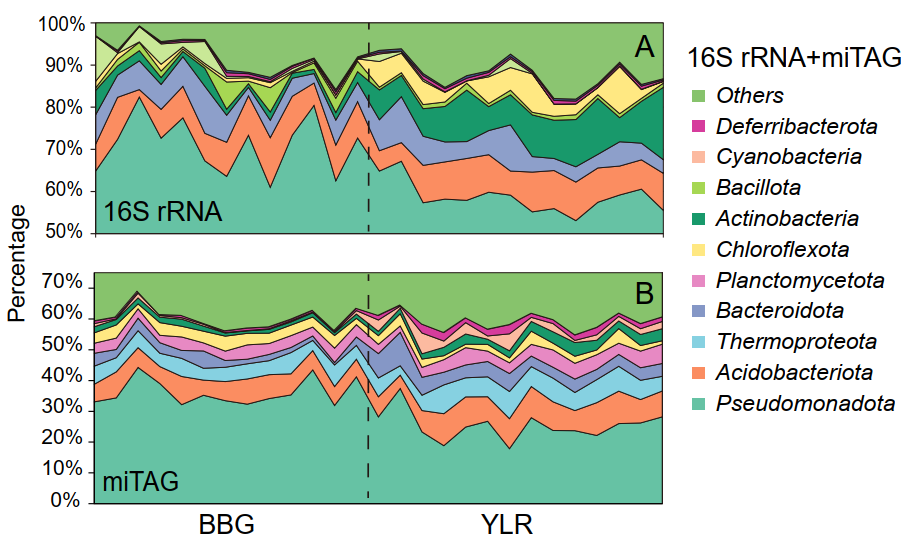


Supplementary Figure 3. Relative abundances of (A) 16S rRNA and (B) miTAG on phylum-level microbial communities between BBG and YLR.


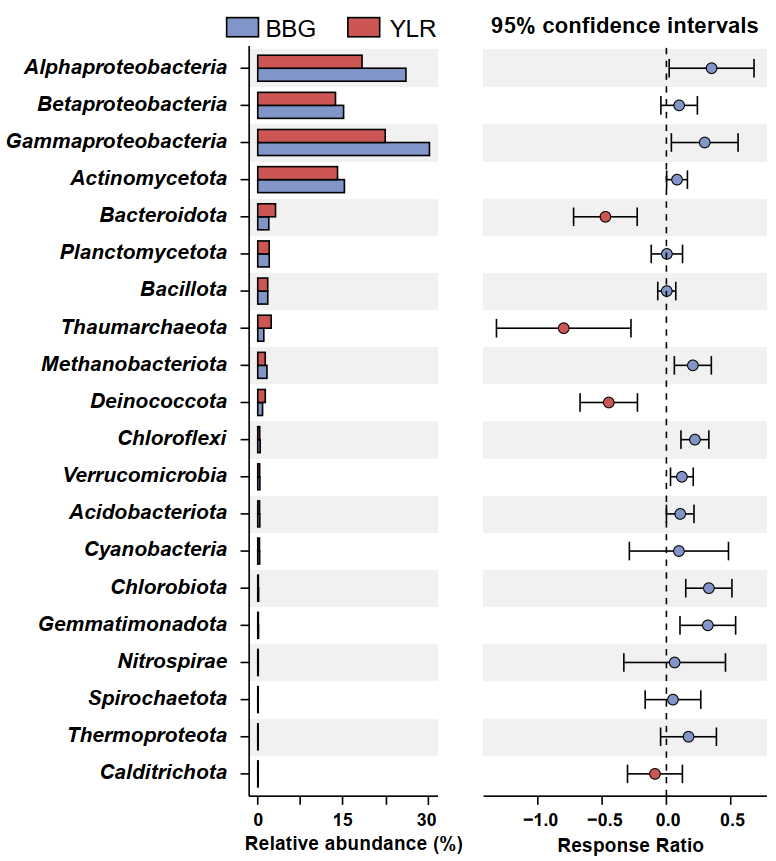


Supplementary Figure 4. The mean relative abundance and response ratio analysis of microbial phyla (class-level for *Pseudomonadota*) between in BBG and YLR. The mean relative abundance of taxonomic groups was shown in the left panel, and the response ratio of taxonomic groups between in BBG and YLR was shown in the right panel. Points represented mean response ratios and error bars were drawn at the 95% confidence interval.


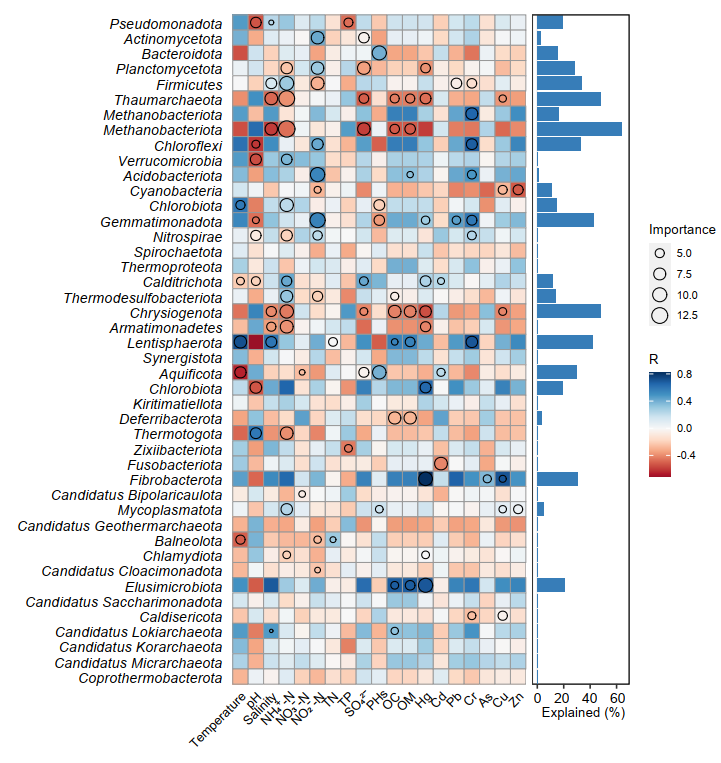


Supplementary Figure 5. The association of environmental factors and N-cycling microbial communities in BBG and YLR. Microbial phyla involved in N-cycling processes were analyzed. Heatmap referred to the associations between the relative abundance of microbial phyla and environmental factors, while the circle size indicated the importance of environmental factors in explaining the variations of individual taxa by random forest analysis (in the left panel); in the right panel, bar plots represented that explained variations by the best environmental factor (the one with the largest circle).

1. **Supplementary tables**

Supplementary Table 3. The partial Mantel test describes the effects of environmental factors on the microbial composition of N-cycling functional traits and taxonomic groups between BBG and YLR.

|  | **Taxonomy** | | | **Function** | |
| --- | --- | --- | --- | --- | --- |
|  | **R** | | ***P*** | **R** | ***P*** |
| Temperature | | 0.175031 | 0.049 | 0.057362 | 0.291 |
| pH | | 0.132890 | 0.039 | 0.206798 | 0.013 |
| Salinity | | -0.0352 | 0.659 | 0.095960 | 0.117 |
| NH_4_^+^-N | | 0.143881 | 0.108 | 0.184848 | 0.058 |
| NO_3_^-^-N | | -0.00478 | 0.484 | -0.01874 | 0.552 |
| NO_2_^-^-N | | 0.026080 | 0.371 | -0.07404 | 0.764 |
| TN | | -0.02139 | 0.539 | 0.032853 | 0.367 |
| TP | | 0.174752 | 0.064 | 0.064115 | 0.24 |
| SO_4_^-^ | | 0.236783 | 0.005 | 0.411162 | 0.001 |
| PHs | | 0.217506 | 0.015 | 0.146941 | 0.045 |
| OC | | 0.030609 | 0.359 | 0.217965 | 0.014 |
| OM | | 0.029561 | 0.373 | 0.217263 | 0.016 |
| Hg | | -0.03351 | 0.563 | 0.096250 | 0.197 |
| Cd | | 0.068243 | 0.243 | 0.163083 | 0.063 |
| Pb | | 0.208370 | 0.068 | 0.271937 | 0.023 |
| Cr | | -0.02024 | 0.558 | 0.026552 | 0.399 |
| As | | 0.124778 | 0.152 | 0.242091 | 0.027 |
| Cu | | -0.02437 | 0.524 | 0.179267 | 0.062 |
| Zn | | 0.026649 | 0.365 | 0.227731 | 0.015 |
